# Mapping Stress, Anxiety, Depression, and Repetitive Negative Thinking Among Non‐Western Undergraduate Students: A Network Analysis

**DOI:** 10.1002/pchj.70043

**Published:** 2025-07-29

**Authors:** Ka Yan, Nessa Ikani, Cleoputri Yusainy, Melissa G. Guineau, Cilia Witteman, Jan Spijker

**Affiliations:** ^1^ Behavioural Science Institute Radboud University Nijmegen the Netherlands; ^2^ Fakultas Psikologi Universitas Kristen Maranatha Bandung Indonesia; ^3^ Depression Expertise Center, Pro Persona Institute for Integrated Mental Health Care Nijmegen the Netherlands; ^4^ Overwaal, Center of Expertise for Anxiety, Obsessive‐Compulsive, and Posttraumatic Stress Disorders, Pro Persona Institute for Integrated Mental Health Care Nijmegen the Netherlands; ^5^ Department of Developmental Psychology, Tilburg School of Social and Behavioral Sciences Tilburg University Tilburg the Netherlands; ^6^ Psychology Department Faculty of Social and Political Sciences Brawijaya University Malang Indonesia

**Keywords:** anxiety, depression, general stress, network analyses, non‐western, RNT, students

## Abstract

Most studies on stress have primarily focused on Western, educated, industrialized, rich, and democratic samples, which may differ from populations in non‐Western countries in terms of how they think and respond to stress. This study investigated the interplay of stress‐related variables, including repetitive negative thinking (RNT), neuroticism, mindful awareness, cognitive control, academic or general stress, anxiety, and depression among Indonesian university undergraduates. Network analyses (association, graphical least absolute shrinkage and selection operator (*g*LASSO), and relative importance network) were conducted to estimate associations between the aforementioned constructs in 474 undergraduate students in Indonesia. Consistent with the association network, the *g*LASSO network revealed that general stress and anxiety had the strongest partial association. The relative importance network further demonstrated that general stress and anxiety exhibited the most robust bidirectional predictive relationships. Furthermore, general stress, RNT, and depression emerged as the strongest predictors within the network structure. The centrality indices from the *g*LASSO network (expected influence, strength, and closeness) identified general stress as the most central node in terms of expected influence and strength. Additionally, RNT and depression showed high strength and closeness values. Similarly, in the relative importance network, RNT, depression, and stress showed the highest outstrength and closeness centrality values. These findings suggest that general stress, anxiety, depression, and RNT are interconnected constructs that play crucial roles in the mental health of non‐Western students. Further studies are required to investigate interventions for those constructs tailored to undergraduate students.

## 1 Introduction

Stress is a common phenomenon among undergraduate students, who may experience ongoing or excessive stress related to academic matters (academic stress) or issues unrelated to their studies (general stress), which can challenge them in maintaining a healthy study‐life balance. A recent international study involving nine countries or regions (Australia, Belgium, Germany, Hong Kong, Mexico, Northern Ireland, South Africa, Spain, and the United States) found that the majority of students reported mild to very severe stress in one or more life areas (e.g., finances, health, relationships; Karyotaki et al. 2020). Additionally, several studies (e.g., Babicka‐Wirkus et al. 2021) showed that the COVID‐19 pandemic is associated with increased levels of distress in students globally. Such prolonged or high levels of stress may lead to the development or exacerbation of (comorbid) anxiety and/or depressive disorders (Khan and Khan 2017).

While mental health challenges are increasingly prevalent among undergraduate students, research is predominantly conducted in Western, educated, industrialized, rich, and democratic (WEIRD) countries (Kotera et al. 2022). This focus limits our understanding of how intercultural differences in mental health are experienced and addressed. For example, people in Eastern countries tend to hold more stigma regarding mental health problems, which impacts not only the individual but also their families. As a consequence, stigmatization is suggested to further decrease mental health and quality of life (Birtel and Mitchell 2023). Compared to Western cultures, which emphasize independence, individuals from Eastern cultures are more rooted in their relationships, reflecting a social orientation based on interdependence. Social involvement is vital as it provides essential support, yet the pressure to uphold these relationships can also increase vulnerability to stress (Lee et al. 2023). Such findings underscore the importance of conducting research in non‐WEIRD countries to ensure broad applicability. Therefore, expanding the scope of research beyond stress, anxiety, and depression to include other psychological constructs provides a more comprehensive understanding of mental health experiences and addresses the unique needs of diverse populations.

In conjunction with this broader perspective, this study covers emotional, cognitive, and personality factors related to stress regulation. To be more specific, we drew on the impaired disengagement framework (De Raedt and Koster 2010; de Lissnyder et al. 2010; Hoorelbeke et al. 2015), which posits that cognitive control processes such as inhibition and attentional shifting may become compromised under stress. This impairment fosters persistent attention to negative stimuli, reinforcing maladaptive evaluative thinking patterns. Furthermore, the response style theory (Nolen‐Hoeksema 1991) and neurotic cascade model (Suls and Martin 2005) emphasize neuroticism's role as a key vulnerability factor. Neuroticism amplifies stress reactivity and contributes to a chain of processes, such as increased negative appraisal, heightened emotional reactivity, and maladaptive coping, that sustain psychological distress. It has also been associated with a greater tendency to engage in repetitive negative thinking (RNT), a mechanism that can reinforce symptoms of anxiety and depression. In the neurotic cascade framework, neuroticism is seen as a dynamic vulnerability rather than a fixed trait, intensifying cognitive and emotional reactivity to stress and influencing how individuals process and respond to challenging experiences. In contrast, mindfulness is theorized to enhance adaptive emotional regulation by promoting non‐judgmental awareness and attentional openness (Brown and Ryan 2003), which may interrupt the negative feedback loop associated with neuroticism and RNT. Having outlined how these constructs are theoretically linked, each is described in greater detail below.

RNT is defined as a thinking style in which negative themes are iteratively rehearsed (Ehring and Watkins 2008; Spinhoven et al. 2018). It can also be perceived as a cognitive habit (Hertel 2015) in response to stress in order to solve emotional problems, understand discrepancies between desired and current (mood) states, down‐regulate stress, or anticipate potential negativity in the future (Anniko 2018; Borkovec et al. 1998; Nolen‐Hoeksema et al. 2008). When excessive, RNT becomes maladaptive, intensifying cognitive inflexibility and exacerbating difficulties in diverting attention away from negative information (Nolen‐Hoeksema et al. 2008). In the academic context, excessive and uncontrollable RNT can heavily impede students' academic performance (Davis 2018) where they may encounter problems concentrating on academic subjects or suffer from a cognitive overload (i.e., trying to process study information while being simultaneously engaged in negative thought loops).

Neuroticism is defined as the dispositional tendency to experience negative emotions, such as anger, anxiety, self‐consciousness, impatience, and guilt (Widiger and Oltmanns 2017). Neuroticism has been significantly associated with elevated vulnerability to stress, anxiety, and depression (Purnamasari and Cahyani 2019; Shokrkon and Nicoladis 2021), and also RNT (Segerstrom et al. 2000). People who are high in neuroticism are prone to getting frustrated easily and tend to be more sensitive to trivial problems or punitive cues, and they often perceive a situation as stressful or threatening (Barnhofer and Chittka 2010). This, in turn, may make students feel more stressed, anxious, or depressed, which, eventually, when faced with continued academic challenges, may cause them to be overwhelmed by negative thoughts (Perkins et al. 2015).

In contrast to neuroticism, mindful awareness is coined as a trait that may serve to protect against the effects of prolonged stress, anxiety, depression, and RNT (i.e., Petrocchi and Ottaviani 2016; Polizzi et al. 2018; Raes and Williams 2010; Vargas‐Nieto et al. 2022). Mindful awareness is thought to alter the way people connect to present‐moment experiences by neutrally reperceiving them (i.e., bringing about a shift from a self‐centered to an objective perspective; Josefsson et al. 2017). By nonjudgmentally observing one's emotions, mindful awareness increases one's ability to regulate negative emotions and to respond in a nonreactive manner. As such, it induces the belief that negative experiences will pass, which promotes acceptance and could counteract RNT (Josefsson et al. 2017; Kong et al. 2014). Hence, mindful awareness has been shown to mitigate neuroticism (Polizzi et al. 2018) and RNT by actively recognizing worry or ruminative thoughts and decentering from them before they become uncontrollable (Raes and Williams 2010). Particularly, students reporting high(er) levels of mindful awareness show better cognitive functioning (e.g., Firth et al. 2023; Lee 2023; Li et al. 2021), suggesting that being mindful may aid academic performance.

Relatedly, cognitive control is theorized to aid in stress regulation and in dealing with anxiety and depressive symptoms (e.g., Gabrys et al. 2018; König et al. 2021). Cognitive control is described as a limited‐capacity resource needed to help one focus on relevant information about immediate goals while ignoring irrelevant information (Gabrys et al. 2018). However, stress, anxiety, and depression, along with RNT and neuroticism, are linked to more difficulties in exerting cognitive control (e.g., Hoorelbeke et al. 2015; LeMoult et al. 2020; Liao et al. 2019). When cognitive control is impaired, RNT is more likely to dominate people's minds in stressful situations (Hoorelbeke et al. 2016), which in turn can lead to or exacerbate feelings of anxiety and depression (Snyder and Hankin 2016). Similarly, individuals reporting high levels of neuroticism have been found to experience more difficulties in shifting attention away from negative information compared to more neutral information. Consequently, individuals tend to be preoccupied with negative thoughts, which can disturb the accomplishment of tasks, particularly those requiring cognitive control (Robison et al. 2017). Thus, limited cognitive control can impede students' learning and concentration. A growing body of research (e.g., Grundy et al. 2018; Hirsch and Mathews 2012; Liao et al. 2019; Valadez 2015) indeed demonstrates that RNT and neuroticism impair cognitive control, whereas mindful awareness is suggested to promote it. Mindfulness may increase cognitive control by maintaining attention on the present task and minimizing emotional distraction (Grundy et al. 2018). In keeping with this, mindfulness training has been proven to increase cognitive control (Larson et al. 2013) and reduce RNT (Denis 2021; Raes and Williams 2010), stress (Palmer and Rodger 2009), anxiety, and depressive symptoms (Parmentier et al. 2019). Similar findings have also been found in randomized control trials examining the effects of mindfulness‐based cognitive therapy on anxiety and depression (MBCT, e.g., Nandarathana and Ranjan 2024).

It is worth noting that these constructs were predominantly developed in Western contexts (Barnhofer and Chittka 2010; Ehring and Watkins 2008; Gabrys et al. 2018; Nolen‐Hoeksema et al. 2008). As previously mentioned, cultural context plays an important role in how individuals experience and regulate their thoughts, emotions, and behavior. Hamamura et al. (2009) described how “approach–avoidance motivations”‐the tendency to pursue desirable outcomes (approach) or avoid undesirable ones (avoidance)—influence cognitive processes. Individuals in WEIRD countries are more driven by approach motivation (such as self‐esteem), whereas individuals in non‐WEIRD contexts, such as Indonesia, often demonstrate a strong desire to avoid negative outcomes, such as shame or embarrassment from failing to meet others' expectations. Maintaining social harmony is important in Indonesian society, making individuals often hide personal issues as private (Putri et al. 2022). This aligns with many Eastern cultures that favor emotional restraint, which has been linked to self‐critical thinking (Raphiphatthana et al. 2019) and emotional suppression (Keng et al. 2017). These culturally ingrained habits of self‐criticism may contribute to RNT. Additionally, the pressure to conform to strong social norms has been associated with higher levels of neuroticism in Eastern contexts (Ibrahim 1979). Mindfulness, on the other hand, promotes non‐judgmental awareness and acceptance (Josefsson et al. 2017). This may become an adaptive alternative as it eases the impact of ruminative thinking within these cultural environments (Karl et al. 2022). Similarly, cognitive control might be beneficial for adaptation, as it enables individuals to manage relational obligations and uphold social cohesion. It often involves regulating emotional expressions and ensuring that one's behavior aligns with social norms (Gavazzi et al. 2023; Matthews et al. 2021). Thus, cultural factors do not alter the function of cognitive control per se, but rather shape its adaptive deployment in socially meaningful ways.

Despite ample studies investigating the associations among stress, anxiety, depression, RNT, neuroticism, mindful awareness, and cognitive control (e.g., Armstrong and Rimes 2016; Hernández‐Torrano et al. 2020; Liao et al. 2019), these variables were mostly studied in isolation (e.g., the link between anxiety and neuroticism). There is no study that systematically mapped them together, despite theoretical frameworks suggesting that they interact with each other. Moreover, contradictory results have been found. For example, a study using UK Biobank data found no association between stress, depression, or suicide and neuroticism (Batty et al. 2016), while another study from a related sample reported the opposite findings, identifying neuroticism as a factor that increases suicide risk (Peters et al. 2018). Ganesan et al. (2024) conducted an 8‐week cognitive control training on academic achievement in students, along with a 1‐year follow‐up, and found no significant effects. Thus, no strong evidence was found showing that increased cognitive control could serve as a buffer for mental health issues. Further research is needed to understand the interplay of stress, anxiety, depression, and related factors, as stress‐related matters arise from complex interactions of individual and social factors.

We adopted network analysis as this method best allows for the analysis and visualization of complex relationships and structures among constructs within a network (Borsboom and Cramer 2013; Bringmann et al. 2019). Network analysis offers a different approach to investigating psychological variables as it treats them as interconnected constructs that interact with each other, and not as variables resulting from a latent construct, as is the case in traditional analytical approaches (Jones and Robinaugh 2021; Robinaugh et al. 2016). This perspective offers insight into the central role of particular variables and how they connect to other variables in the network. Although network analysis is primarily viewed as an exploratory method, it offers valuable understanding of which variables can be targeted for the intervention and to enhance the effectiveness of treatment protocols (Zagaria et al. 2023). Given the cultural differences that we mentioned above, we may find potential different patterns between stress‐related constructs than those observed in WEIRD countries through network analysis. For example, in cultural contexts where individuals are encouraged to suppress mental health concerns to maintain social harmony, this suppression may contribute to elevated levels of anxiety and RNT—particularly in collectivistic societies where emotional restraint is valued (Keng et al. 2017; Cheung and Park 2010). Consequently, RNT and anxiety may emerge as more central in the network structure, potentially altering their relationships with other variables and highlighting them as key intervention targets.

The importance of this study is further highlighted by previous studies revealing the high prevalence of stress, anxiety, and depression in adolescents and young adults in Indonesia (e.g., El‐Matury et al. 2018; Marthoenis et al. 2018), a country with a culture that differs considerably from most Western countries. However, most studies investigating stress among students in Indonesia are limited to its links with anxiety and depression. Given that many psychological disorders emerge during young adulthood (Ferrari et al. 2022), broadening the scope of research to include the various factors described above is essential, reinforcing this study's relevance. Therefore, we aimed to investigate the interaction between academic and general stress, anxiety, depressive symptoms, RNT, neuroticism, mindful awareness, and cognitive control among undergraduate students in Indonesia. We hypothesized that academic stress, general stress, anxiety, depression, RNT, and neuroticism would have positive associations, while mindfulness would have a positive association with cognitive control. Moreover, academic stress, general stress, anxiety, depression, RNT, and neuroticism were expected to have negative associations with both mindfulness and cognitive control. Since this study was conducted during the COVID‐19 pandemic, we also took the situation into account.

## 2 Methods

### 2.1 Recruitment and Participants

Participants were recruited through advertisement posts on social media, which included information about the study and eligibility criteria. Eligible participants were undergraduate students currently enrolled at a university in Indonesia. To ensure the representativeness of the sample, we invited participants from the western, central, and eastern parts of Indonesia, particularly from Java (central Indonesia), given that most universities are concentrated there. Participants received study credits or proportional financial compensation for their participation. The study was reviewed and approved by the ethics committee of Universitas Kristen Maranatha.

### 2.2 Instruments

#### 2.2.1 Academic Stress

The Academic Stress Inventory (ASI; Lin and Chen 2009) includes 34 items assessing study‐related stress. Using a five‐point Likert scale (1 = *strongly disagree* to 5 = *strongly agree*), its seven subscales gauge stress associated with teachers, results, tests, studying in groups, peers, time management, and self‐inflicted stress. Internal consistency was excellent in the current sample (α = 0.918).

#### 2.2.2 Depression, Anxiety and Stress

The Depression, Anxiety and Stress Scales (DASS‐21; Lovibond and Lovibond 1995) includes a total of 21 items to be rated on a four‐point Likert scale (0 = *did not apply to me at all* to 3 = *applied to me very much or most of the time*), generating a total score and three subscale scores. In the current research, the internal consistency of the total scale score (α = 0.927), and the scores for the depression subscale (α = 0.878), the anxiety subscale (α = 0.807) and stress subscale (α = 0.825) were all high.

#### 2.2.3 Trait RNT

To look for the presence of (the trait of) RNT, the Perseverative Thinking Questionnaire (PTQ; Ehring et al. 2011) was administered. It includes 15 items to be rated on a five‐point Likert scale (0 = *never* to 4 = *almost always*). The scale showed excellent internal consistency in the current sample (α = 0.949).

#### 2.2.4 Neuroticism

The Big Five Inventory‐Neuroticism (BFI‐N; John et al. 1991) was used and consists of eight items that are rated on a five‐point Likert scale (1 = *totally disagree* to 5 = *totally agree*). The internal consistency of this inventory was good in the current sample (α = 0.806).

#### 2.2.5 Mindful Awareness

The Mindful Attention Awareness Scale (MAAS; Brown and Ryan 2003) was used to gauge mindful awareness using 15 items rated on a six‐point Likert scale (1 = *almost always* to 6 = *almost never*). In this study, the internal consistency was good (α = 0.855).

#### 2.2.6 Cognitive Control

The Effortful Control scale of the Adult Temperament Questionnaire (ATQ‐short form; Evans and Rothbart 2007) was administered. It includes 19 items that have to be rated on a seven‐point Likert scale (1 = *extremely untrue of you* to 7 = *extremely true of you*). The subscale's internal consistency in this study was acceptable (α = 0.727).

#### 2.2.7 COVID‐19 Questions

We formulated four items to specifically test any (additional) stress experienced during and related to the COVID‐19 pandemic, that is, stress due to studying online, (the lack of) peer interaction, financial problems, and family relations, all rated on a seven‐point Likert scale (1 = *not stressful at all* to 7 = *very stressful*). Cronbach's alpha in our study was 0.671, indicating that the internal consistency was considered fairly acceptable.

Six questionnaires were used, of which three (i.e., DASS‐21, BFI‐N, MAAS) were already available in Bahasa Indonesia, the official national language of Indonesia, and had been validated (Nada et al. 2022; Ramdhani 2012; Yusainy et al. 2018). The other three (ASI, PTQ, ATQ) were translated into Bahasa specifically for this study. A team of experts consisting of academics who are also psychologists from Universitas Kristen Maranatha, with extensive experience in the diagnosis and treatment of clinical/mental health issues and a professional interpreter fluent in English and Bahasa, translated, back translated, and checked the final Bahasa versions of the questionnaires.

### 2.3 Procedures

Participants who responded to the online advertisements were asked to provide informed consent prior to their participation and to complete the online questionnaires via Qualtrics. The total survey took approximately 30 min to complete.

### 2.4 Statistical Analysis

We opted for a network approach as it is a robust methodology to evaluate complex relationships among multiple psychological constructs (Borsboom and Cramer 2013). The results can be described visually, using nodes (variables) and edges (symbolized by lines representing the associations among variables; Epskamp et al. 2018). The thickness of the edges indicates the strength of the relationships between/among nodes (Pulopulos et al. 2022), which can be either positive (green line) or negative (red line). All analyses were conducted in the R programming environment (Version 3.6.3; R Core Team 2020). There are eight nodes in this study: academic stress, general stress, anxiety, depression, RNT, neuroticism, mindful awareness, and cognitive control. We did not include COVID‐19 questions in the network analysis, as those items were used solely for contextual purposes and were not part of our theoretical or statistical model.

### 2.5 General Analysis Routine

#### 2.5.1 Sample Size Estimation

A simulation study by Epskamp (2016) illustrated that 250 participants are needed to achieve a median sensitivity (i.e., proportion of edges in the true network that were found to be non‐zero) of ~0.60 in a network of 25 nodes. In this study, the network consisted of 8 nodes. Therefore, a sample of *N* = 250 was considered to provide sufficient sensitivity. To complement this, a sample size estimation was conducted using the *powerly* package in R (Constantin et al. 2023), which applies a general Monte Carlo approach for power analysis in psychological network models. We performed 10,000 simulation runs using the bootstrapped splines method, assuming medium‐sized effects. The results indicated that a sample of 226 participants would be sufficient to detect stable edges under conditions similar to those in this study.

#### 2.5.2 Estimation Method

We involved three types of networks: association, graphical least absolute shrinkage and selection operator (*g*LASSO), and relative importance. First, an association network (using the *qgraph* package) was computed to estimate the structure of the network, with the edges representing zero‐order correlations. The weights of the edges indicate the strength of the relationships between nodes (Robinaugh et al. 2016). Second, a *g*LASSO network was computed using the R packages *qgraph* (Epskamp et al. 2012) and *glasso* (Friedman et al. 2008). The resultant network represents a regularized partial correlation network and estimates unique associations between nodes, thereby limiting potentially false positives or spurious associations by shrinking small associations to zero (Epskamp et al. 2018). A tuning parameter of 0.25 was selected for the EBICglasso model, as it provides a moderately conservative estimate by balancing the removal of spurious edges with the retention of meaningful associations (Epskamp et al. 2017; Hevey 2018; Guineau et al. 2023). This approach enhances both the specificity and interpretability of the resulting network structure (Epskamp et al. 2017; Epskamp and Fried 2018).

Third, using the *Relaimpo* package, a relative importance network was computed to determine the contribution that each node makes to the explained variance (*R* ^2^) after controlling for the effects of other nodes in the network. Each node's contribution to R^2^ is quantified by a relative importance metric (*lmg*), which ranges from 0 to 1 (Guineau et al. 2023). A relative importance network is weighted, and the predictive strength and directionality of edges are represented by two arrows that may have different thicknesses because of non‐symmetrical associations (Bringmann et al. 2019).

#### 2.5.3 Accuracy and Stability of Edge‐Estimates

To ensure the reliability of interpretations, the stability of the obtained centrality indices of the *g*LASSO and relative importance network were separately assessed using the R package *bootnet* (Epskamp et al. 2018). The 95% confidence intervals (CIs) for the edges were calculated using a non‐parametric 1000‐sample bootstrap technique. The resulting correlation stability (CS) coefficient should be above 0.25 but preferably larger than 0.5 to be considered stable.

### 2.6 Analysis‐Specific Routine

#### 2.6.1 Centrality Indices

To analyze the magnitude of the associations and determine the structural importance of nodes in the *g*LASSO network, centrality indices of betweenness, closeness, strength (Bringmann et al. 2019), and expected influence (EI) were used (Robinaugh et al. 2016). Betweenness indicates how frequently a node lies on the shortest path between two nodes, closeness indicates the inverse of the sum describing how often a node is indirectly connected to other nodes, strength refers to the absolute value describing the edge weights with which a node is directly connected to other nodes, and EI is the sum of a node's connections while taking into account the sign of an edge (Robinaugh et al. 2016).

Similar to the other networks, centrality indices in a relative importance network were also obtained. However, unlike the others, a distinction is made between in‐strength and out‐strength. In‐strength denotes the extent to which a given node is influenced by other nodes, and out‐strength quantifies the extent to which a given node influences other nodes, with higher values reflecting greater centrality in this respect (Bringmann et al. 2019).

Furthermore, we conducted a bootstrapped difference test to assess whether centrality values significantly differed between specific nodes, using the *bootnet* package in R.

#### 2.6.2 Differences Between Edges Within the Network

A bootstrapped difference test was performed using the *bootnet* package to examine whether certain edge weights significantly differed from one another, indicating which connections were stronger or more prominent in the network (Epskamp et al. 2018).

## 3 Results

### 3.1 General Analysis Routine

#### 3.1.1 Final Sample Size

A total of 479 undergraduate students completed the survey. Multivariate outliers were detected using Mahalanobis distance (Sapp et al. 2007). Accordingly, five participants were removed, resulting in an analytical dataset of 474 participants (382 female and 92 male, Mean_age_ = 20.11, SD_age_ = 2.84). This exclusion targeted extreme cases (*p* < 0.001) to ensure data quality while maintaining a robust sample size (see Table 1 for the descriptive data).

**TABLE 1:** Descriptives of all variables of the sample.

	*Mean*	SD	Min	Max
Academic stress (ASI)	90.03	21.26	40.00	146.00
Stress (DASS‐21 stress subscale)	18.35	8.54	0.00	42.00
Anxiety (DASS‐21 anxiety subscale)	15.88	8.47	0.00	42.00
Depression (DASS‐21 depression subscale)	14.19	9.07	0.00	42.00
Repetitive negative thinking (PTQ)	30.51	11.95	0.00	60.00
Neuroticism (BFI‐N)	26.50	5.63	8.00	39.00
Trait mindful awareness (MAAS)	57.61	11.35	25.00	89.00
Cognitive control (ATQ‐short form)	84.81	12.46	41.00	125.00
Covid‐19 items				
Online learning	4.54	1.37	1	7
Relations with friends	4.10	1.68	1	7
Economic situation	4.26	1.85	1	7
Relations with family	3.16	1.87	1	7

*Note*: ‘Stress’ refers to general stress (i.e., stress experienced in daily life, outside of academia).

Abbreviation: SD = standard deviation.

No missing data were present in the dataset. As *g*LASSO estimates a Gaussian Graphical Model, it assumes multivariate normality. To examine this, we inspected a Q–Q plot of the Mahalanobis distance (Naik 2003), which revealed that the data is normally distributed. Therefore, no data transformations were applied. This approach is consistent with current practices in similar psychological network research (e.g., Bringmann et al. 2019; Epskamp and Fried 2018; Haslbeck and Waldorp 2018; van Borkulo et al. 2023).

#### 3.1.2 Results of Accuracy and Stability Checks

Bootstrapped CIs for the *g*LASSO and relative importance networks were relatively narrow, indicating stable results (see Figures S2 and S6, Supporting Information).

### 3.2 Analysis‐Specific Routine

#### 3.2.1 Network Visualization

**FIGURE 1:**
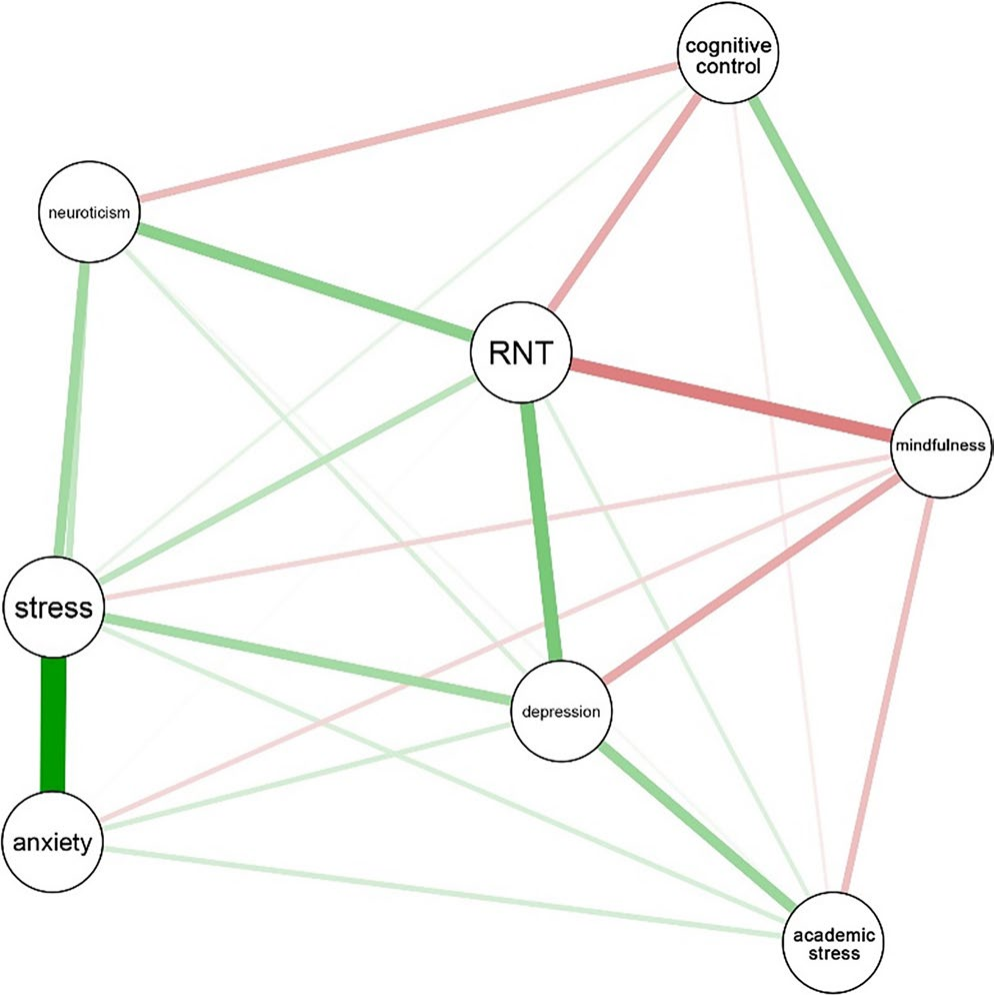
Graphical LASSO network. Every variable is represented by a node, with each edge signifying a regularized partial correlation between two nodes. A green line indicates a positive association while a red line denotes a negative association, and the thickness of an edge reflects the magnitude of the association. ‘Stress’ refers to general stress (i.e., stress experienced in daily life, outside of academia) and ‘mindfulness’ connotes ‘mindful awareness’.

The *g*LASSO network is depicted in Figure 1. We applied the Fruchterman–Reingold force‐directed layout algorithm, which spatially arranges nodes based on simulated repulsion and attraction forces. This method promotes visual interpretability by reducing node overlap and creating evenly spaced edges (Fruchterman and Reingold 1991; Jones et al. 2018).

Consistent with the correlation matrix of the association network (see Table S1), the *g*LASSO network also revealed that general stress and anxiety had the strongest association (*pr* = 0.513), followed by depression and RNT (*pr* = 0.270), and RNT and mindful awareness (*pr* = −0.255). General stress emerged as a node that was connected to all other nodes in the network (See Table S2 for the regularized partial correlation matrix of the *g*LASSO network).

The relative importance network is depicted in Figure 3. The relative importance values (i.e., *lmg*) are presented in Table S4 in the Supporting Information. General stress and anxiety had the strongest bidirectional relationships. General stress predicted anxiety (*lmg* = 0.41), and anxiety predicted general stress (*lmg* = 0.33). Additionally, RNT predicted mindful awareness (*lmg* = 0.24), depression (*lmg* = 0.22), and general stress (*lmg* = 0.14). Depression (*lmg* = 0.21), mindful awareness (*lmg* = 0.19), and general stress (*lmg* = 0.15) also predicted RNT. Accordingly, RNT and depression, RNT and mindful awareness, and stress and RNT showed a bidirectional relationship, while a symmetrical bidirectional relationship was found between depression and anxiety (*lmg* = 0.14). The analyses hence showed that general stress, RNT, and depression were the strongest predictors.

#### 3.2.2 Centrality Indices

##### 3.2.2.1 Graphical LASSO Network

**FIGURE 2:**
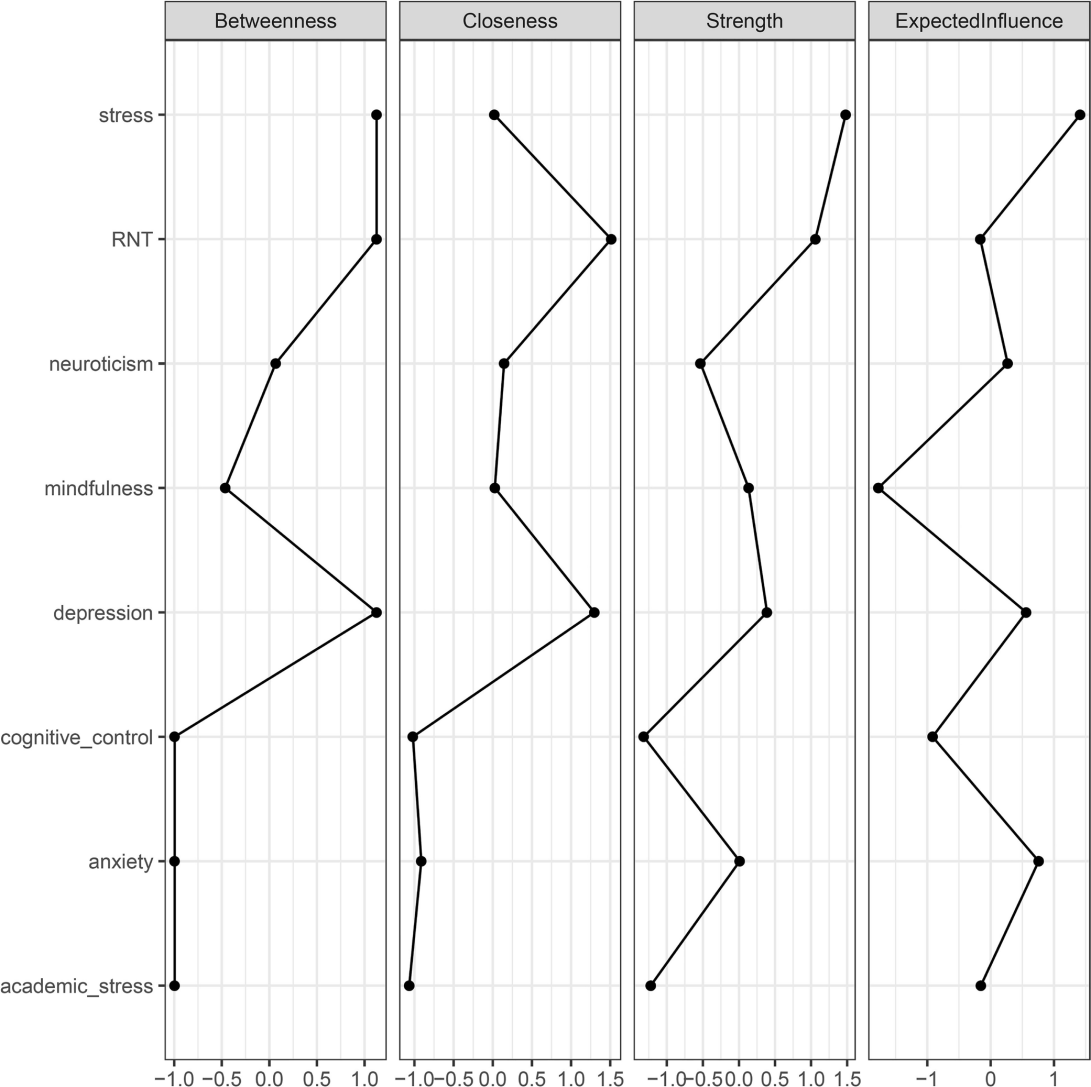
Plot of centrality indices *of* gLASSO. ‘Stress’ refers to general stress (i.e., stress experienced in daily life, outside of academia) and ‘mindfulness’ connotes ‘mindful awareness’.

The CS‐coefficients for EI, strength, and closeness were 0.751, 0.673, and 0.517, respectively, indicating strong stability for these indices. In contrast, the CS‐coefficient for betweenness was 0.205, falling below the recommended cut‐off (Epskamp et al. 2018), and therefore not considered reliable (see Figure S1). Accordingly, we focus the interpretations on EI, strength, and closeness centrality, which meet the standards for robustness. General stress showed the highest level of EI, followed by anxiety and depression. General stress, RNT, and depression showed the highest level of strength. RNT and depression showed high levels of closeness (see Figure 2 and Table S3 for centrality indices for all nodes of the gLASSO network in the Supporting Information). Furthermore, to assess the centrality rankings, nonparametric bootstrapped difference tests for node strength, closeness, and expected influence were conducted (Figure S3a–c). Accordingly, based on EI, stress was significantly more central than depression, neuroticism, academic stress, RNT, cognitive control, and mindfulness. Based on strength, stress was significantly more central than RNT, depression, mindfulness, and anxiety. Based on closeness, RNT was more central than neuroticism, anxiety, cognitive control, and academic stress.

**FIGURE 3:**
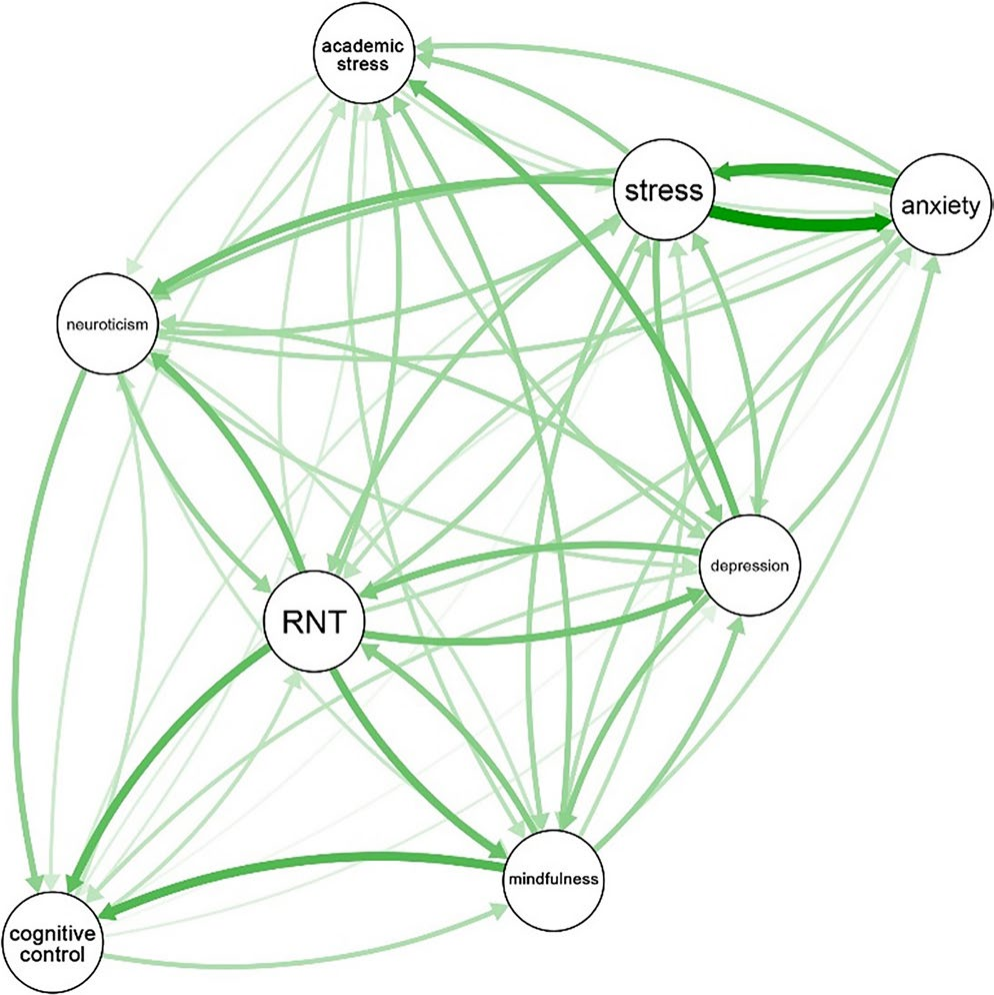
Relative importance network. All edges denote the relative importance of a variable as a predictor of other variables, with arrows indicating the direction of the prediction. ‘Stress’ refers to general stress (i.e., stress experienced in daily life, outside of academia) and ‘mindfulness’ connotes ‘mindful awareness’.

**FIGURE 4:**
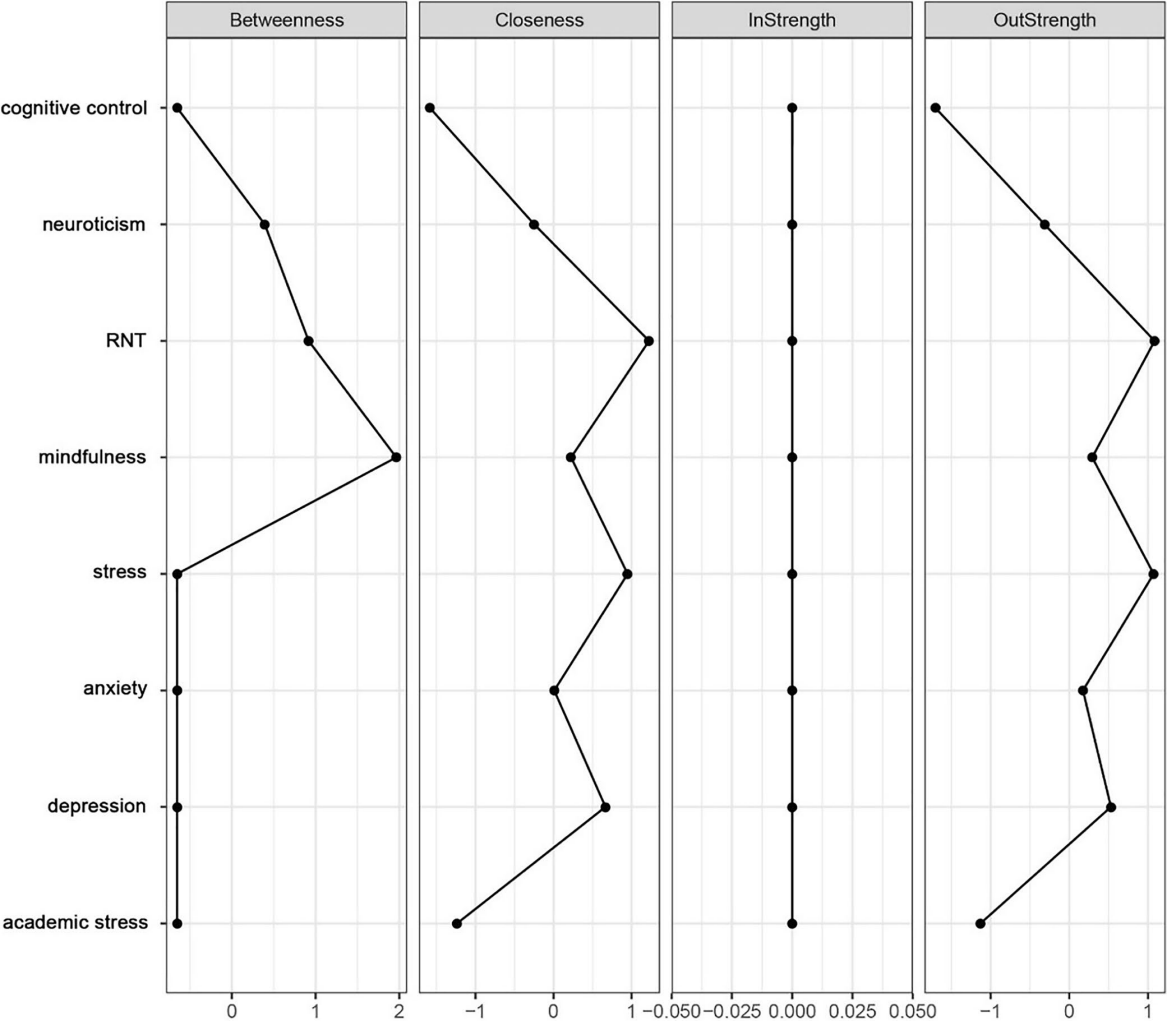
Plot of centrality indices for relative importance network. ‘Stress’ refers to general stress (i.e., stress experienced in daily life, outside of academia) and ‘mindfulness’ connotes ‘mindful awareness’.

##### 3.2.2.2 Relative Importance Network

The CS‐coefficients for out‐strength (0.75) and closeness (0.75) exceeded the recommended threshold, indicating strong stability. In contrast, CS‐coefficients for in‐strength (0.00) and betweenness (0.051) were below the threshold and not interpreted further (see Figure S5 in the Supporting Information). Based on the interpretable indices, RNT, general stress, and depression appeared to be the most central nodes (Figure 4). Furthermore, we tested whether nodes significantly differed in out‐strength (Figure S7a) and closeness (Figure S7b). These results showed that RNT and stress were significantly more central than mindfulness, anxiety, neuroticism, academic stress, and cognitive control. In addition, depression showed significantly higher centrality than neuroticism, academic stress, and cognitive control. See Supporting Information (Tables S4 and S5, Figure S7a,b) for the correlation matrix and centrality within the relative importance network, and the bootstrapped results for out‐strength and closeness.

#### 3.2.3 Specific Edges

Bootstrapped edge difference tests for both the *g*LASSO and relative importance networks revealed that the edge between stress and anxiety was the strongest in the network (*p* < 0.05), highlighting its central role within the network structure (see Figures S4 and S8 in the Supporting Information).

## 4 Discussion

Mental health challenges among undergraduate students are on the rise globally. However, much existing research continues to rely on samples from WEIRD populations (Kotera et al. 2023), limiting generalizability. Furthermore, psychological problems such as stress, anxiety, and depression are often examined in isolation, without considering their dynamic interrelations with cognitive and personality factors. This study addressed these gaps by examining how academic and general stress, anxiety, depression, RNT, neuroticism, mindfulness, and cognitive control interact in a non‐WEIRD sample of Indonesian undergraduate students. We employed three complementary network approaches (an association network, a *g*LASSO network, and a relative importance network) to map the complex structure of these interrelated variables. By identifying the strongest associations and the most central nodes, this study provides insight into the interplay of emotional distress, personality, and cognitive regulation within this population.

The findings showed that general stress and anxiety had the strongest connections across all three networks tested (association, *g*LASSO, and relative importance networks). General stress had the greatest overall EI and strength in the *g*LASSO network. Its connection with anxiety was consistently stronger than any other in the network. This was supported by findings from the relative importance network, showing that general stress and anxiety exhibited the strongest bidirectional relationship. General stress was also identified as a stronger predictor of anxiety and depression than the reverse. These findings are in line with prior research and show that general stress often acts as a precursor to these conditions (e.g., Burtscher et al. 2022; De Kloet et al. 2005). From a cultural perspective, individuals from non‐WEIRD countries, such as Indonesia, are often expected to maintain harmony and fulfill social expectations (Hamamura et al. 2009), which may involve the use of emotional suppression (Keng et al. 2017) to deal with mental health issues. Although this strategy is often acceptable and less maladaptive in Eastern societies (Song et al. 2024), it may limit help‐seeking and intensify stress, increasing the risk of anxiety and depression.

Interestingly, when comparing general to academic stress, general stress had stronger associations with almost all other nodes (*g*LASSO network), and its predictive value was also higher (relative importance network) than academic stress. General stress encompasses major life events that extend beyond the scope of academic stress (Wu et al. 2020), which may explain its general consistent presence and potentially cumulative influence. This broader scope of general stress likely amplifies its impact and contributes to stronger associations with variables such as anxiety and depression. In contrast, academic stress is often tied to specific periods of heightened activity (e.g., exam weeks), which makes its influence more situational and less pervasive. Moreover, it may also contribute to or be perceived as part of broader general stress. Additionally, the symmetrical bidirectional relationship observed between anxiety and depression reflects their high comorbidity (Kalin 2020) and highlights how the presence of either one of these symptom clusters can increase the risk of developing the other symptom clusters (Cramer et al. 2010; Groen et al. 2020).

RNT showed high levels of closeness centrality, indicating its important involvement in the network. Furthermore, RNT, general stress, and depression play key roles in the relative importance networks, signifying that changes in these variables are more likely to bring about a change in the network structure. As such, they serve as predictors of other nodes. More specifically, general stress and RNT demonstrated a bidirectional relationship. RNT is widely recognized as a transdiagnostic risk factor for both the development and maintenance of affective disorders (Ehring and Watkins 2008), and stress is theorized to exacerbate this vulnerability (Stamatis et al. 2020). Furthermore, RNT appears to prolong the impact of stressors by maintaining event‐related cognitions long after the stressor has occurred (Anniko 2018). Eastern countries often stigmatize mental health issues, which can lead to negative reactions from others and even from individuals themselves. This internalized stigma can trigger negative self‐perceptions and emotions (Kudva et al. 2020). As a result, it may also contribute to the role of RNT in worsening distress.

RNT's prominent role in the network is consistent with prior findings identifying it as a key vulnerability factor for depression (e.g., Nolen‐Hoeksema 1991). On the other hand, depressive symptoms may also contribute to increased RNT, as individuals prone to depression tend to have negative cognitive biases (Bottemanne et al. 2022; Paul et al. 2013), including a consistent focus on negative stimuli or mood (Rood et al. 2010). In Eastern cultural contexts, heightened sensitivity to failure and self‐criticism (Raphiphatthana et al. 2019; Hamamura et al. 2009) may contribute to feelings of helplessness, which are central to depression. Once depressive symptoms emerge, they may further exacerbate RNT, reinforcing the depression–RNT cycle and prolonging psychological distress. Furthermore, in both the association and *g*LASSO networks, mindful awareness was negatively linked to RNT. Instead of being present in the moment, individuals with a tendency for excessive RNT focus on negative thoughts and irrelevant information (Gustavson et al. 2018), such as preoccupation with judgment and social evaluation, which may be shaped by social norms around self‐monitoring and social evaluation. Eventually, this may make it difficult for them to remain present and nonjudgmental and diminish mindful awareness.

The network analyses also reveal several interesting findings that contrast with the general literature derived from WEIRD samples (e.g., Gabrys et al. 2018; Gustavson et al. 2018). The associations between cognitive control and general stress, anxiety, and depression were weak across all networks, which suggests that cognitive control exerted minimal influence on these constructs. An explanation, however, might be that our self‐report measurement of cognitive control did not adequately reflect cognitive control ability. Indeed, it has been suggested that self‐reports represent one's *belief* in their cognitive control ability instead (e.g., Todd et al. 2022). Future studies should, therefore, incorporate validated cognitive control tasks to obtain a more accurate measurement of it. Another explanation could be related to cultural differences. In non‐WEIRD countries, individuals may prioritize conflict avoidance and group harmony over direct emotion regulation strategies, such as cognitive appraisal (Song et al. 2024). This is possibly contributing to the limited role of cognitive control observed in the network.

Furthermore, a very weak association was observed between RNT and anxiety in the *g*LASSO network (*pr* = 0.008), suggesting that these variables were only minimally related after controlling for all other nodes. This finding contrasts with previous studies reporting a stronger association between RNT and anxiety (e.g., Gustavson et al. 2018). A potential explanation for this discrepancy is the nature of the measures used. That is, the DASS‐21 anxiety subscale primarily captures physical symptoms related to anxiety, whereas RNT reflects a more cognitive response style (Ehring and Watkins 2008; Nolen‐Hoeksema 1991).

The *g*LASSO network showed no correlation between mindful awareness and neuroticism. This discrepancy may be explained by the critical role of RNT in linking mindfulness and neuroticism, as demonstrated by the *g*LASSO and relative importance networks. The findings align with research and suggest that RNT may act as a mediator in psychological distress (e.g., Macedo et al. 2015; Peixoto and Cunha 2022). As described earlier, in non‐WEIRD contexts, self‐critical tendencies are common, and emotional restraint is often encouraged (Hamamura et al. 2009; Keng et al. 2017). Therefore, RNT may evolve into a habitual coping mechanism (Tichenor et al. 2023). Sustained RNT may also contribute to heightened emotional reactivity and instability in response to distress, which are characteristics associated with neuroticism.

In this sample, neuroticism played a less prominent role in the interplay between academic stress, general stress, anxiety, and depressive symptoms. The relative importance network analyses supported this, which identified neuroticism as one of the weakest predictors, with general stress, RNT, and depression predicting neuroticism but not the reverse. When individuals experience stress, anxiety, or depression, they may adopt maladaptive coping strategies that increase their susceptibility to neuroticism (Hajek et al. 2020). Prolonged exposure to stress may exacerbate neuroticism, as individuals with high neuroticism tend to perceive stress as more threatening and believe they lack the resources to cope with it (Amestoy et al. 2023). This worsens their neurotic tendencies and deepens their psychological vulnerability.

The relative importance network also indicated that depression was more predictive of academic stress than the other way around, a finding that diverges from previous research suggesting that academic stress influences the severity of depressive symptoms (e.g., Chen et al. 2024; Deng et al. 2022). Feelings of helplessness and sadness associated with depressive symptoms can hinder concentration, which makes it harder for students to achieve their academic goals (Ali et al. 2024). Additionally, depressive symptoms often diminish students' interest and motivation for learning, which can undermine their confidence and foster more negative self‐perceptions and make them perceive themselves as incompetent (Awadalla et al. 2020). Furthermore, their tendency to be preoccupied with and dwell on negative thoughts (Rood et al. 2010) can further impair their ability to manage academic stress, thereby exacerbating the impact on their academic performance. In Eastern cultures, students may internalize the notion that academic success is tied to social status and family expectations (Ali et al. 2019). As a result, students who feel helpless—a common feature of depression—may perceive academic demands as overwhelming, which can increase their levels of academic stress.

Indonesian undergraduates in the current study reported high levels of general stress, anxiety, and depressive symptoms. These elevated ratings may have been affected by the extraordinary circumstances of the COVID‐19 pandemic. Many participants noted that online education, changes in peer interactions, and financial challenges substantially increased their levels of stress (see Table 1). However, prior research in Indonesia demonstrated that even before the COVID‐19 pandemic, students reported higher levels of general stress, anxiety, and depression (see El‐Matury et al. 2018) compared to undergraduates in WEIRD countries (e.g., Bibi et al. 2020; Ramón‐Arbués et al. 2020). Similar findings were observed in other non‐WEIRD populations (e.g., Asif et al. 2020; Panda et al. 2017). These findings shine a light on the vital role of cultural differences when studying mental health and underscore the need for cross‐cultural research to better understand the mental health of young adults globally. In our study, the strong relationships among general stress, anxiety, depression, and RNT suggest these constructs are culturally relevant intervention targets in the Indonesian student population.

The results of this study have to be interpreted within the context of several limitations. First, while the MAAS is a validated instrument, it predominantly focuses on self‐awareness and may not fully capture other important aspects of mindful awareness (Zhuang et al. 2017).

Further studies should replicate the networks covering other measures, like the Five‐Facet Mindful Awareness Questionnaire (FFMQ; Zhuang et al. 2017), in which other dimensions of mindful awareness are covered. This could provide a more comprehensive understanding of how different components of mindful awareness relate to stress, anxiety, or depression. Second, we relied on convenience sampling, with women comprising 80% of the sample. As women are generally more vulnerable to stress, anxiety, and depression (e.g., Özdin and Özdin 2020), this gender imbalance limits the generalizability. Third, although prior research suggests that the overall structure of psychological networks, such as symptom connectivity, can be relatively stable across studies, specific quantitative properties like node centrality or edge strength are more sensitive to sample characteristics and may not replicate consistently (Borsboom et al. 2018). For instance, centrality profiles in a nonclinical undergraduate population may differ substantially from those in clinical samples, even within the same culture. Future research should include more balanced and representative samples to ensure that results can be generalized across genders and diverse populations. Fourth, our analyses were based on cross‐sectional data, which precludes any causal conclusions. While the findings indicate interconnected roles of RNT and other constructs, we note that network analysis cannot establish causal mediation.

In sum, this work provides empirical evidence of the interplay of stress‐related constructs through a comprehensive lens of network analysis in a non‐WEIRD country. The present study highlights the pivotal roles of general stress, anxiety, depression, and RNT, which enhance our understanding of mental health dynamics of young adults. It is suggested that future studies investigate interventions specifically tied to these constructs.

## Ethics Statement

All procedures involving human participants were conducted in accordance with the ethical standards of the institutional research committee at Universitas Kristen Maranatha and with the 1964 Helsinki Declaration and its later amendments or comparable ethical standards.

## Consent

Informed consent was obtained from all individual adult participants included in the study.

## Conflicts of Interest

The authors declare no conflicts of interest.

## Supporting information


**Data S1:** Supporting Information.

## Data Availability

The data that support the findings of this study are available from the corresponding author upon reasonable request.
