# Supplementary material for: Mapping Stress, Anxiety, Depression, and Repetitive Negative Thinking Among Non‐Western Undergraduate Students: A Network Analysis
Source: Psych J. 2025 Jul 29;14(5):635–49. doi: 10.1002/pchj.70043 (PMC12520848; doi:10.1002/pchj.70043)
Supplement: Supplementary file 1 — Data S1: Supporting Information. [file PCHJ-14-635-s001.docx]

**Supplementary Material**

**Table S1**

Correlation matrix of association

|  | | | | | | | | | | | | | | | | | | | | | |
| --- | --- | --- | --- | --- | --- | --- | --- | --- | --- | --- | --- | --- | --- | --- | --- | --- | --- | --- | --- | --- | --- |
|  | **Network** | | | | | | | | | | | | | | | | | | | | |
| **Variable** | **Academic**  **Stress** | | | **Depression** | | **Anxiety** | | | **Stres** | | | **Mindfulness** | | **Rnt** | **Neuroticism** | | | | **Cognitive**  **Control** | | |
| Academic stress |  | 0.000 |  | 0.579 |  | 0.514 |  | | 0.543 |  | -0.525 | |  | 0.532 |  | 0.453 |  | -0.340 | |  |  |
| Depression |  | 0.579 |  | 0.000 |  | 0.624 |  | | 0.686 |  | -0.640 | |  | 0.706 |  | 0.581 |  | -0.400 | |  |  |
| Anxiety |  | 0.514 |  | 0.624 |  | 0.000 |  | | 0.788 |  | -0.565 | |  | 0.595 |  | 0.592 |  | -0.306 | |  |  |
| Stress |  | 0.543 |  | 0.686 |  | 0.788 |  | | 0.000 |  | -0.605 | |  | 0.665 |  | 0.640 |  | -0.325 | |  |  |
| Mindfulness |  | -0.525 |  | -0.640 |  | -0.565 |  | | -0.605 |  | 0.000 | |  | -0.678 |  | -0.510 |  | 0.488 | |  |  |
| RNT |  | 0.532 |  | 0.706 |  | 0.595 |  | | 0.665 |  | -0.678 | |  | 0.000 |  | 0.641 |  | -0.494 | |  |  |
| Neuroticism |  | 0.453 |  | 0.581 |  | 0.592 |  | | 0.640 |  | -0.510 | |  | 0.641 |  | 0.000 |  | -0.428 | |  |  |
| Cognitive Control |  | -0.340 |  | -0.400 |  | -0.306 |  | -0.325 | |  | 0.488 | |  | -0.494 |  | -0.428 |  | 0.000 | |  |  |
|  | | | | | | | | | | | | | | | | | | | | | |

‘Stress’ refers to general stress (i.e., stress experienced in daily life, outside of academia) and ‘mindfulness’ connotes ‘mindful awareness.’

**Table S2**

Correlation matrix of *g*LASSO

|  | | **Network** | | | | | | | | | | | | | | |  |
| --- | --- | --- | --- | --- | --- | --- | --- | --- | --- | --- | --- | --- | --- | --- | --- | --- | --- |
| **Variable** | **Academic**  **Stres** | | | **Depression** | | **Anxiety** | | **Stress** | | **Mindfulness** | | **RNT** | **Neuroticism** | | **Cognitive**  **Control** | |  |
| Academic stress |  | 0.000 |  | 0.199 |  | 0.085 |  | 0.077 |  | -0.127 |  | 0.064 |  | 0.025 |  | -0.037 | |
| Depression |  | 0.199 |  | 0.000 |  | 0.083 |  | 0.182 |  | -0.168 |  | 0.270 |  | 0.070 |  | 0.000 | |
| Anxiety |  | 0.085 |  | 0.083 |  | 0.000 |  | 0.513 |  | -0.078 |  | 0.008 |  | 0.121 |  | 0.000 | |
| Stress |  | 0.077 |  | 0.182 |  | 0.513 |  | 0.000 |  | -0.082 |  | 0.130 |  | 0.185 |  | 0.047 | |
| Mindfulness |  | -0.127 |  | -0.168 |  | -0.078 |  | -0.082 |  | 0.000 |  | -0.255 |  | 0.000 |  | 0.205 | |
| RNT |  | 0.064 |  | 0.270 |  | 0.008 |  | 0.130 |  | -0.255 |  | 0.000 |  | 0.230 |  | -0.165 | |
| Neuroticism |  | 0.025 |  | 0.070 |  | 0.121 |  | 0.185 |  | 0.000 |  | 0.230 |  | 0.000 |  | -0.137 | |
| Cognitive control |  | -0.037 |  | 0.000 |  | 0.000 |  | 0.047 |  | 0.205 |  | -0.165 |  | -0.137 |  | 0.000 | |

‘Stress’ refers to general stress (i.e., stress experienced in daily life, outside of academia) and ‘mindfulness’ connotes ‘mindful awareness.’

| **Table S3**  Centrality measures per variable (*g*LASSO) | | | | | | | | | |
| --- | --- | --- | --- | --- | --- | --- | --- | --- | --- |
|  | | **Network** | | | | | | | |
| **Variable** | | **Betweenness** | | **Closeness** | | **Strength** | | **Expected influence** | |
| Academic stress |  | -0.995 |  | -1.066 |  | -1.222 |  | -0.154 |  |
| Depression |  | 1.127 |  | 1.297 |  | 0.390 |  | 0.563 |  |
| Anxiety |  | -0.995 |  | -0.912 |  | 0.011 |  | 0.759 |  |
| Stress |  | 1.127 |  | 0.019 |  | 1.480 |  | 1.415 |  |
| Mindfulness |  | -0.464 |  | 0.026 |  | 0.134 |  | -1.773 |  |
| RNT |  | 1.127 |  | 1.512 |  | 1.060 |  | -0.163 |  |
| Neuroticism |  | 0.066 |  | 0.144 |  | -0.532 |  | 0.270 |  |
| Cognitive control |  | -0.995 |  | -1.020 |  | -1.319 |  | -0.916 |  |
|  | | | | | | | | | |

‘Stress’ refers to general stress (i.e., stress experienced in daily life, outside of academia) and ‘mindfulness’ connotes ‘mindful awareness.’


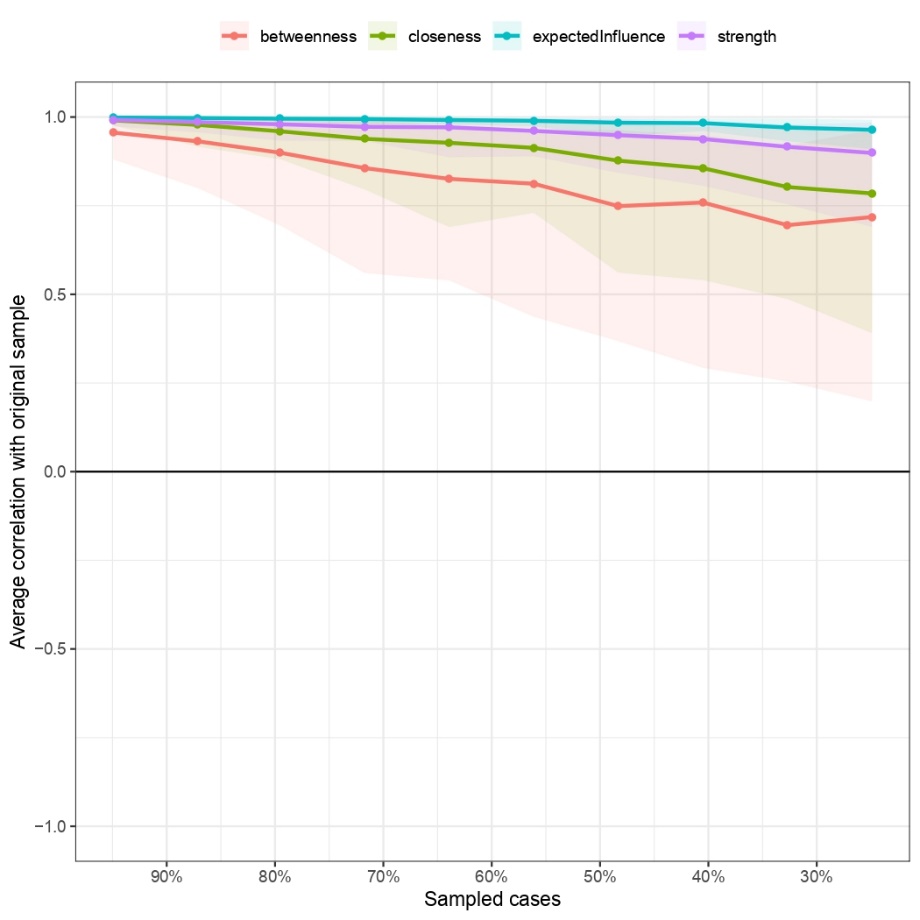


**Figure S1** Case-dropping bootstrap estimates of centrality stability in the *g*LASSO network


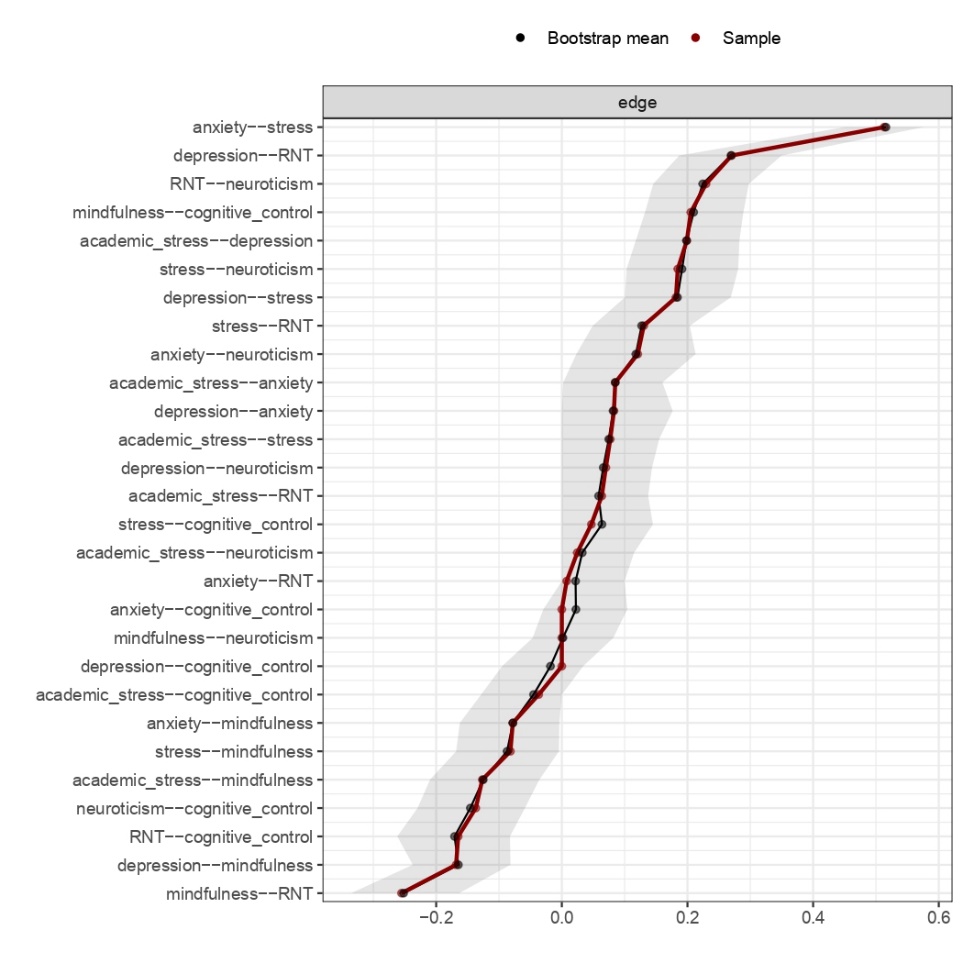


**Figure S2** Bootstrap CIs of *g*LASSO. Note**:** The edge is symbolized by the red line, with the x-axis representing various edges. Along the y-axis, the specific edges are indicated by the grey lines. ‘Stress’ refers to general stress (i.e., stress experienced in daily life, outside of academia) and ‘mindfulness’ connotes ‘mindful awareness.’


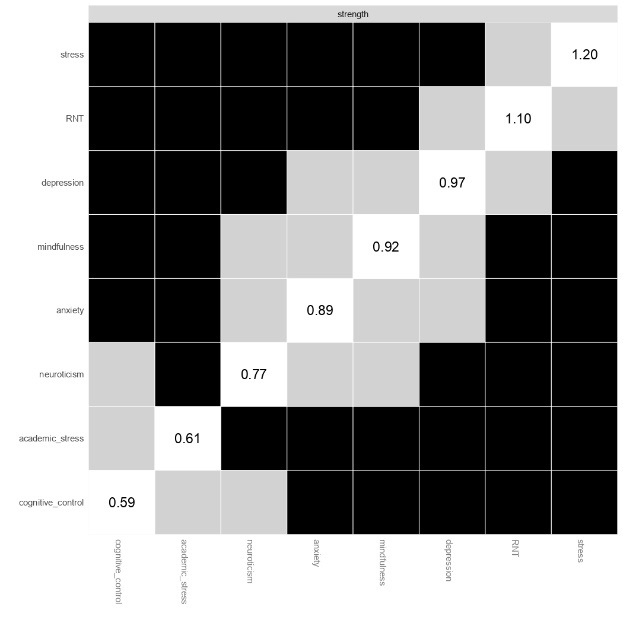


**Figure S3a** Bootstrapped significance test across nodes in the *g*LASSO network**:** Strength. Note: Black boxes represent significant differences from each other. Gray boxes indicate that they do not differ significantly from each other. ‘Stress’ refers to general stress (i.e., stress experienced in daily life, outside of academia) and ‘mindfulness’ connotes ‘mindful awareness.’


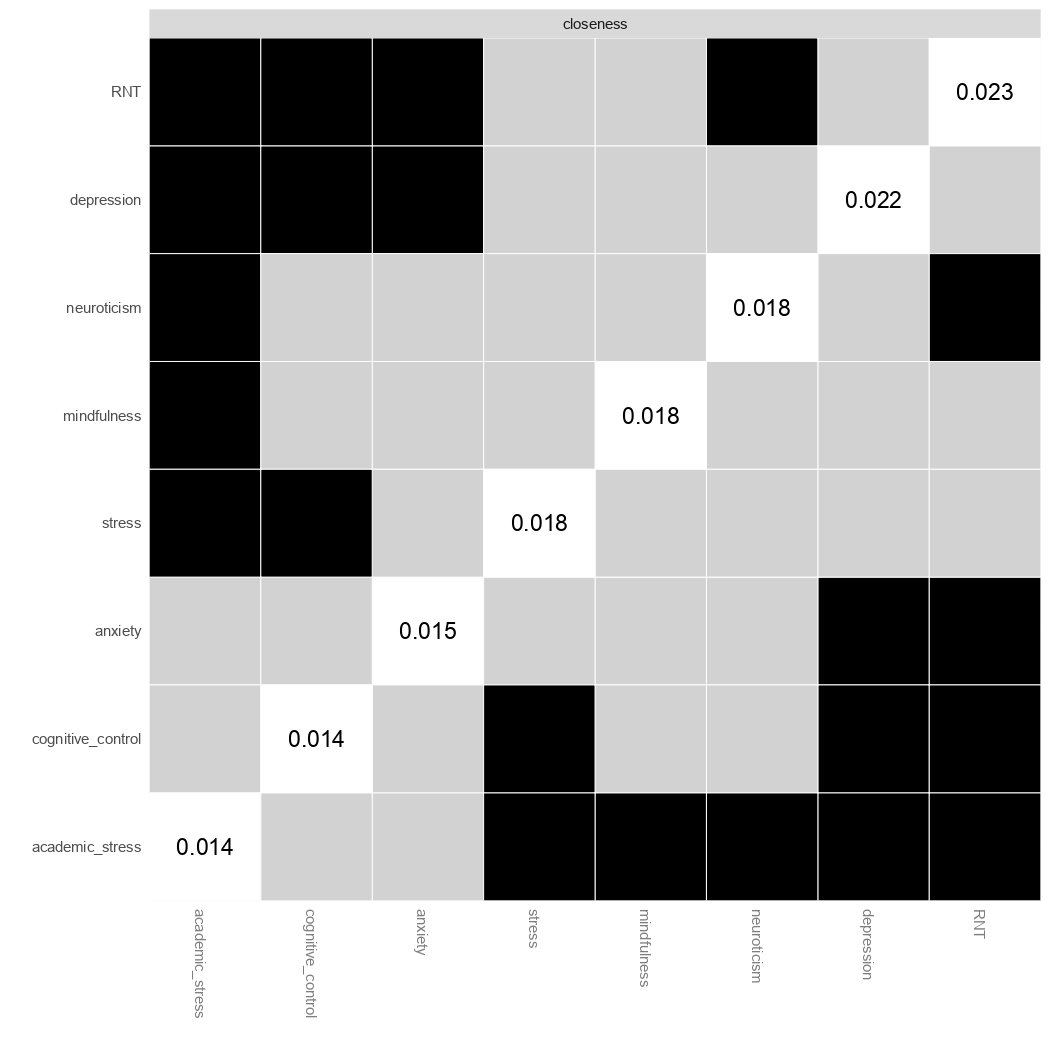


**Figure S3b** Bootstrapped significance test across nodes in the *g*LASSO network**:** Closeness Note: Black boxes represent significant differences from each other. Gray boxes indicate that they do not differ significantly from each other. ‘Stress’ refers to general stress (i.e., stress experienced in daily life, outside of academia) and ‘mindfulness’ connotes ‘mindful awareness.’


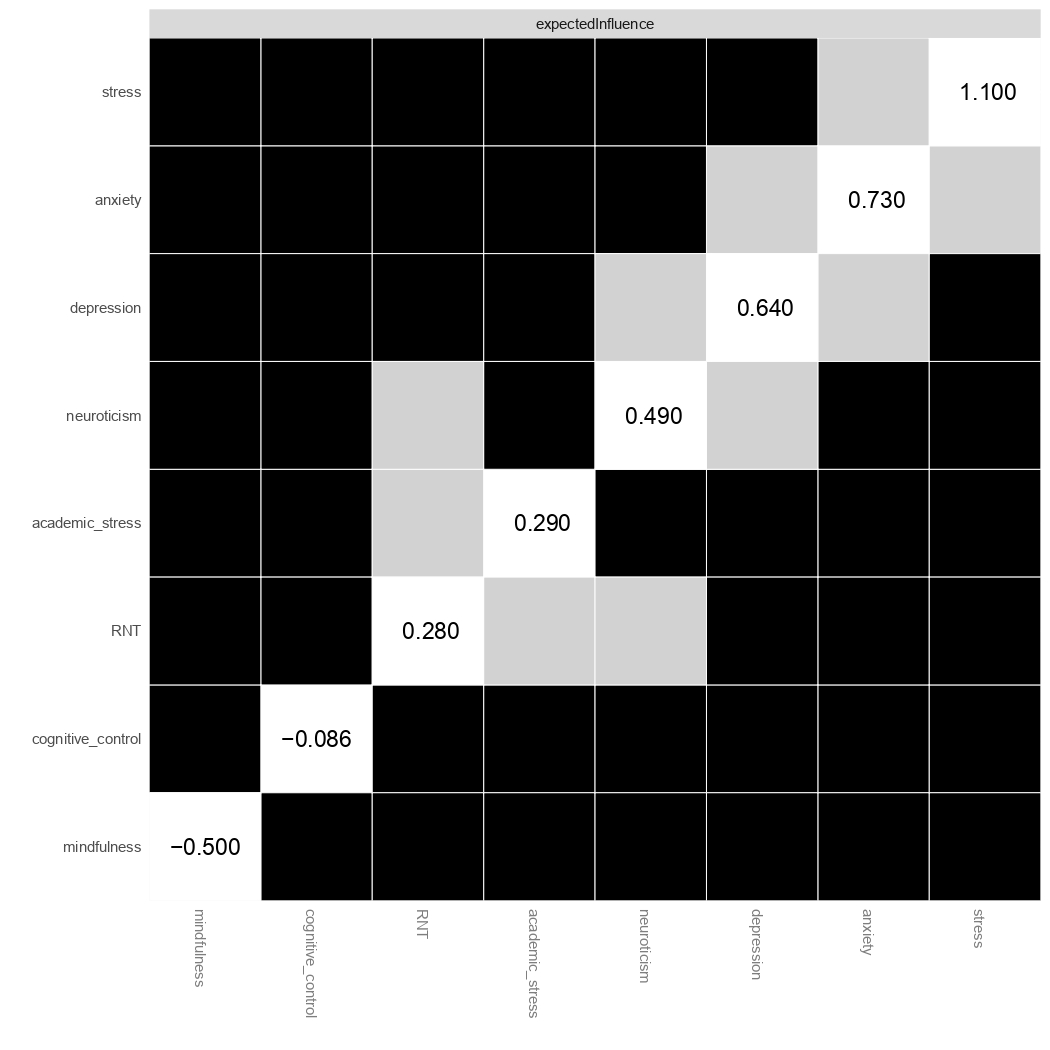


**Figure S3c** Bootstrapped significance test across nodes in the *g*LASSO network**:** Expected Influence. Note: Black boxes represent significant differences from each other. Gray boxes indicate that they do not differ significantly from each other. ‘Stress’ refers to general stress (i.e., stress experienced in daily life, outside of academia) and ‘mindfulness’ connotes ‘mindful awareness’.


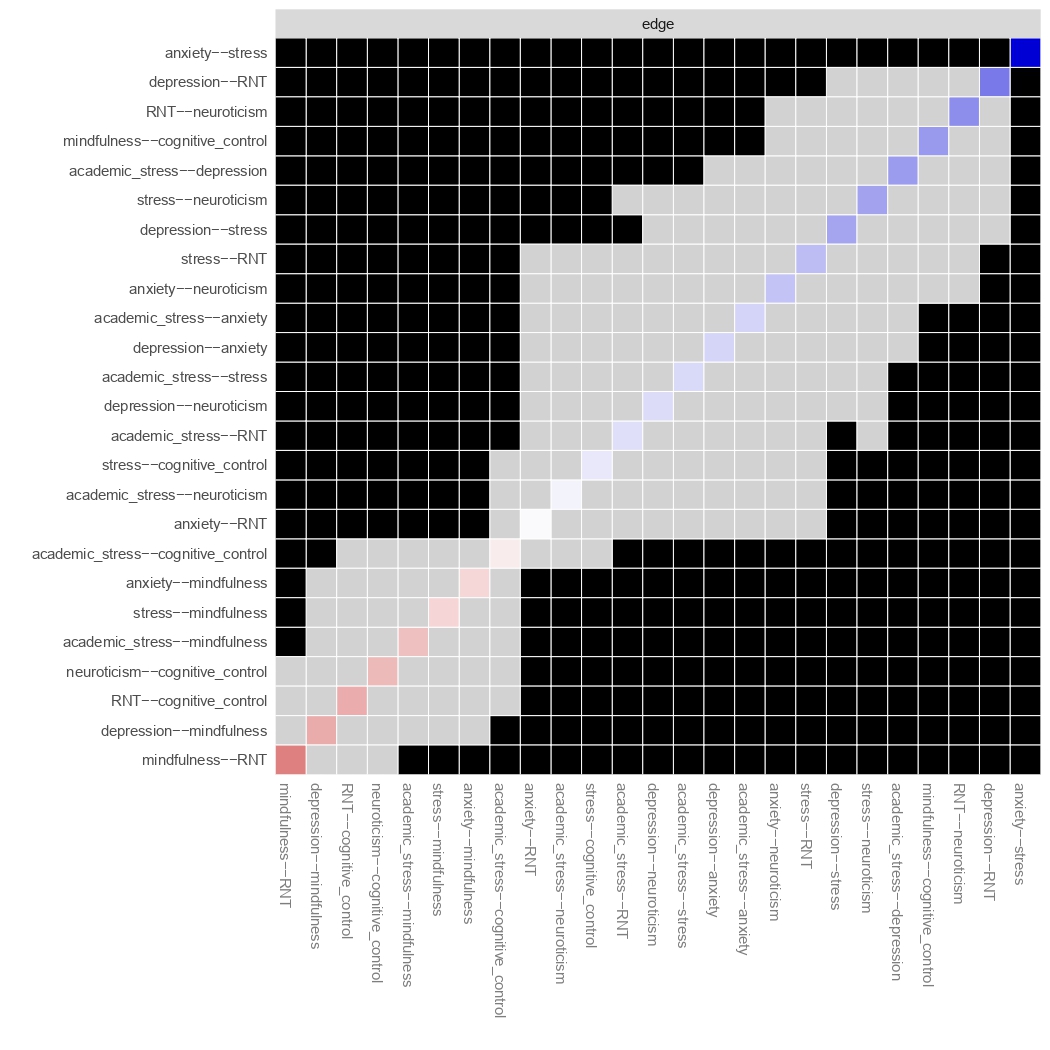


**Figure S4** Bootsrapped significance test of edge weights in the *g*LASSO network. Note: Black boxes represent significant differences from each other. Gray boxes indicate that they do not differ significantly from each other. ‘Stress’ refers to general stress (i.e., stress experienced in daily life, outside of academia) and ‘mindfulness’ connotes ‘mindful awareness.’

**Table S4**

Correlation matrix of the relative importance values

|  | **Network** | | | | | | | |
| --- | --- | --- | --- | --- | --- | --- | --- | --- |
|  | **Academic stress** | **Depression** | **Anxiety** | **Stress** | **Mindfulness** | **RNT** | **Neuroticism** | **Cognitive control** |
| **Academic stress** | 0.000 | 0.133 | 0.086 | 0.085 | 0.109 | 0.085 | 0.071 | 0.075 |
| **Depression** | 0.233 | 0.000 | 0.137 | 0.162 | 0.182 | 0.208 | 0.139 | 0.103 |
| **Anxiety** | 0.144 | 0.137 | 0.000 | 0.334 | 0.119 | 0.106 | 0.165 | 0.048 |
| **Stress** | 0.163 | 0.190 | 0.406 | 0.000 | 0.143 | 0.155 | 0.217 | 0.063 |
| **Mindfulness** | 0.162 | 0.158 | 0.105 | 0.109 | 0.000 | 0.190 | 0.092 | 0.276 |
| **RNT** | 0.149 | 0.223 | 0.112 | 0.142 | 0.237 | 0.000 | 0.222 | 0.255 |
| **Neuroticism** | 0.093 | 0.111 | 0.131 | 0.144 | 0.083 | 0.160 | 0.000 | 0.177 |
| **Cognitive control** | 0.055 | 0.047 | 0.023 | 0.025 | 0.127 | 0.096 | 0.093 | 0.000 |

‘Stress’ refers to general stress (i.e., stress experienced in daily life, outside of academia) and ‘mindfulness’ connotes ‘mindful awareness.’

**Table S5**

Centrality measures per variable (relative importance network)

|  | **Network** | | | |
| --- | --- | --- | --- | --- |
|  | **Out-strength** | **In-Strength** | **Closeness** | **Betweeness** |
| **Academic stress** | 0.645 | 1 | 0.013 | 0 |
| **Depression** | 1.166 | 1 | 0.023 | 0 |
| **Anxiety** | 1.054 | 1 | 0.019 | 0 |
| **Stress** | 1.337 | 1 | 0.024 | 0 |
| **Mindfulness** | 1.092 | 1 | 0.020 | 5 |
| **RNT** | 1.340 | 1 | 0.025 | 3 |
| **Neuroticism** | 0.901 | 1 | 0.018 | 2 |
| **Cognitive control** | 0.465 | 1 | 0.011 | 0 |

‘Stress’ refers to general stress (i.e., stress experienced in daily life, outside of academia) and ‘mindfulness’ connotes ‘mindful awareness.’


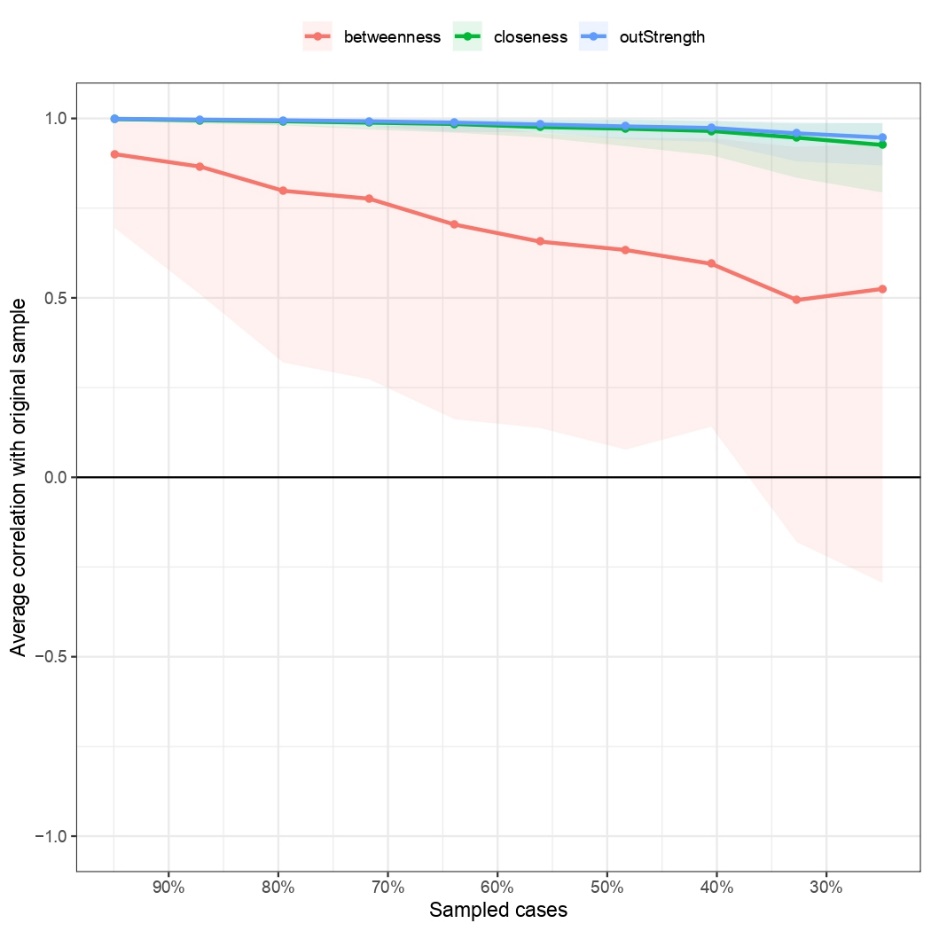


**Figure S5** Case-dropping bootstrap estimates of centrality stability in the relative importance network. Note: In-strength is not displayed, as it shows no variance across nodes.


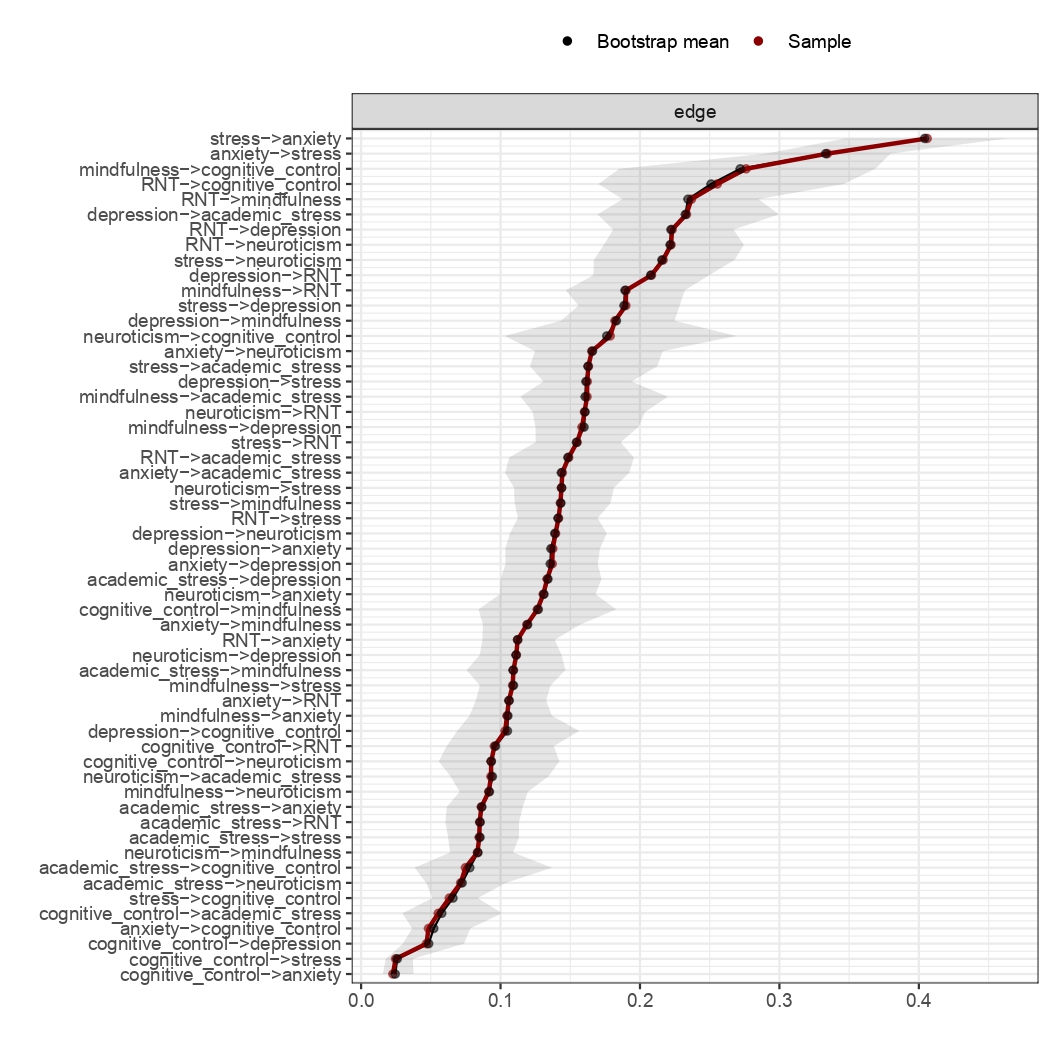


**Figure S6** Bootstrap CIs of relative importance. Note**:** The edge is symbolized by the red line, with the x-axis representing various edges. Along the y-axis, the specific edges are indicated by the grey lines. ‘Stress’ refers to general stress (i.e., stress experienced in daily life, outside of academia) and ‘mindfulness’ connotes ‘mindful awareness.’


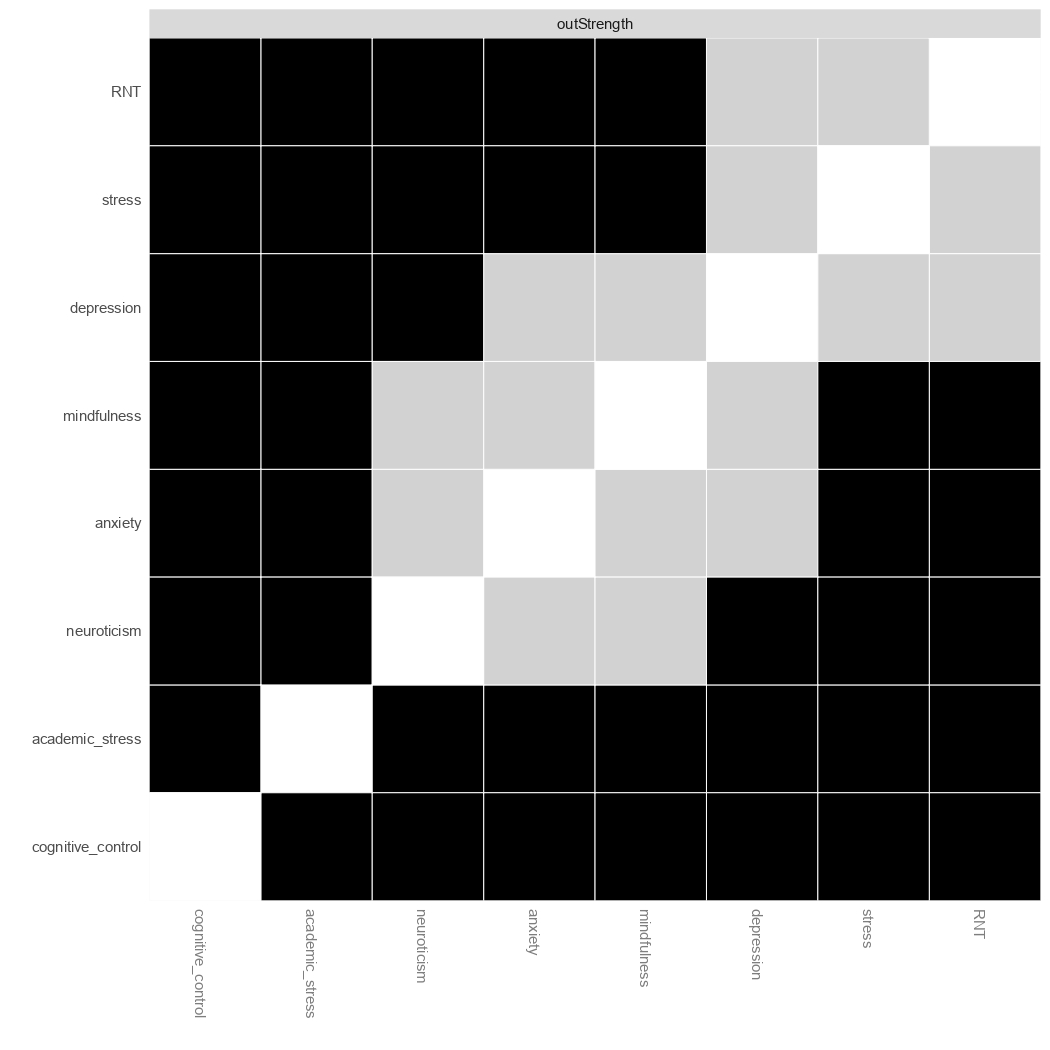


1. (b)

**Figure S7a** Bootstrapped significance test in the relative importance network: Out-strength. Note: A significant difference (p<.05) is represented by a black box at the intersection of a row and a column, while a grey box indicates no significant difference. ‘Stress’ refers to general stress (i.e., stress experienced in daily life, outside of academia) and ‘mindfulness’ connotes ‘mindful awareness.


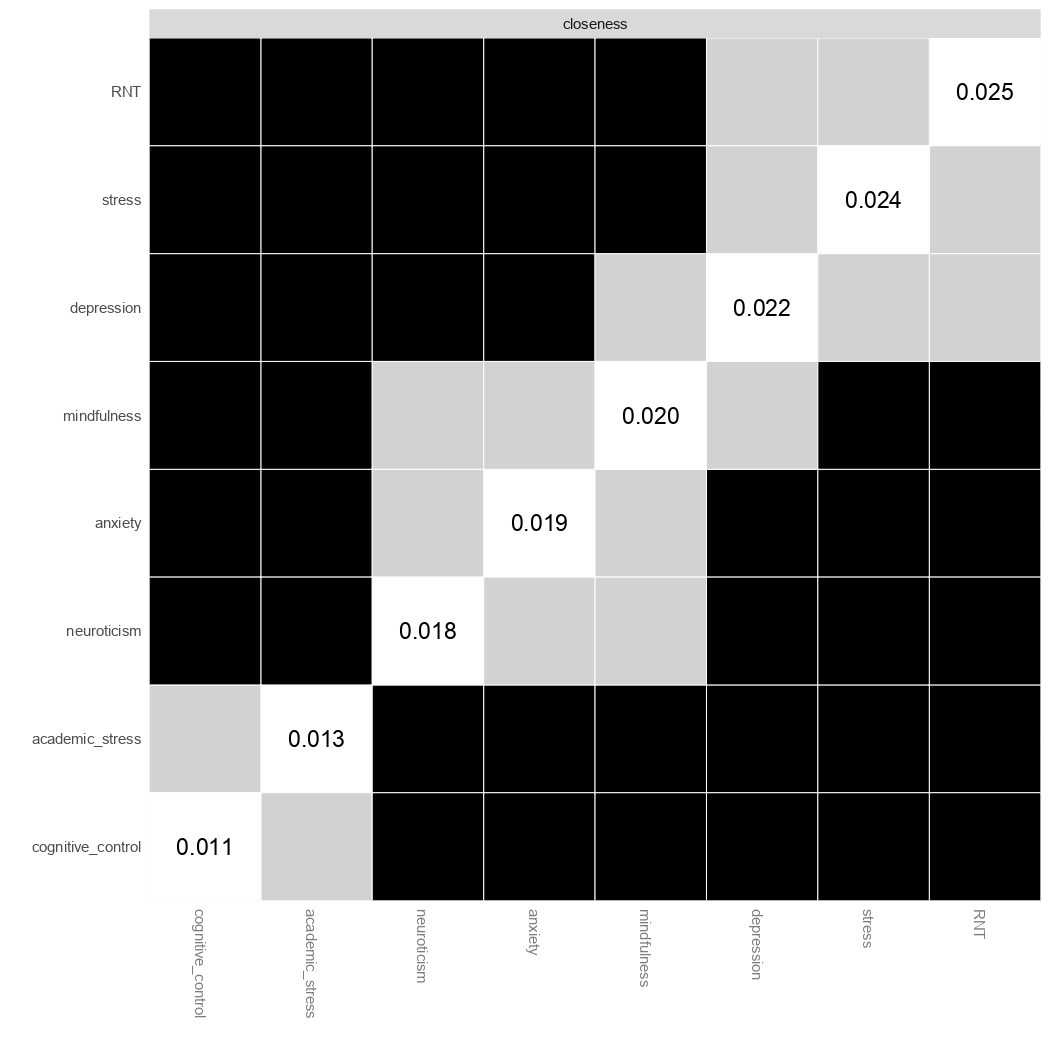


**Figure S7b** Bootstrapped significance test in the relative importance network: Closeness. Note: A significant difference (p<.05) is represented by a black box at the intersection of a row and a column, while a grey box indicates no significant difference. ‘Stress’ refers to general stress (i.e., stress experienced in daily life, outside of academia) and ‘mindfulness’ connotes ‘mindful awareness.


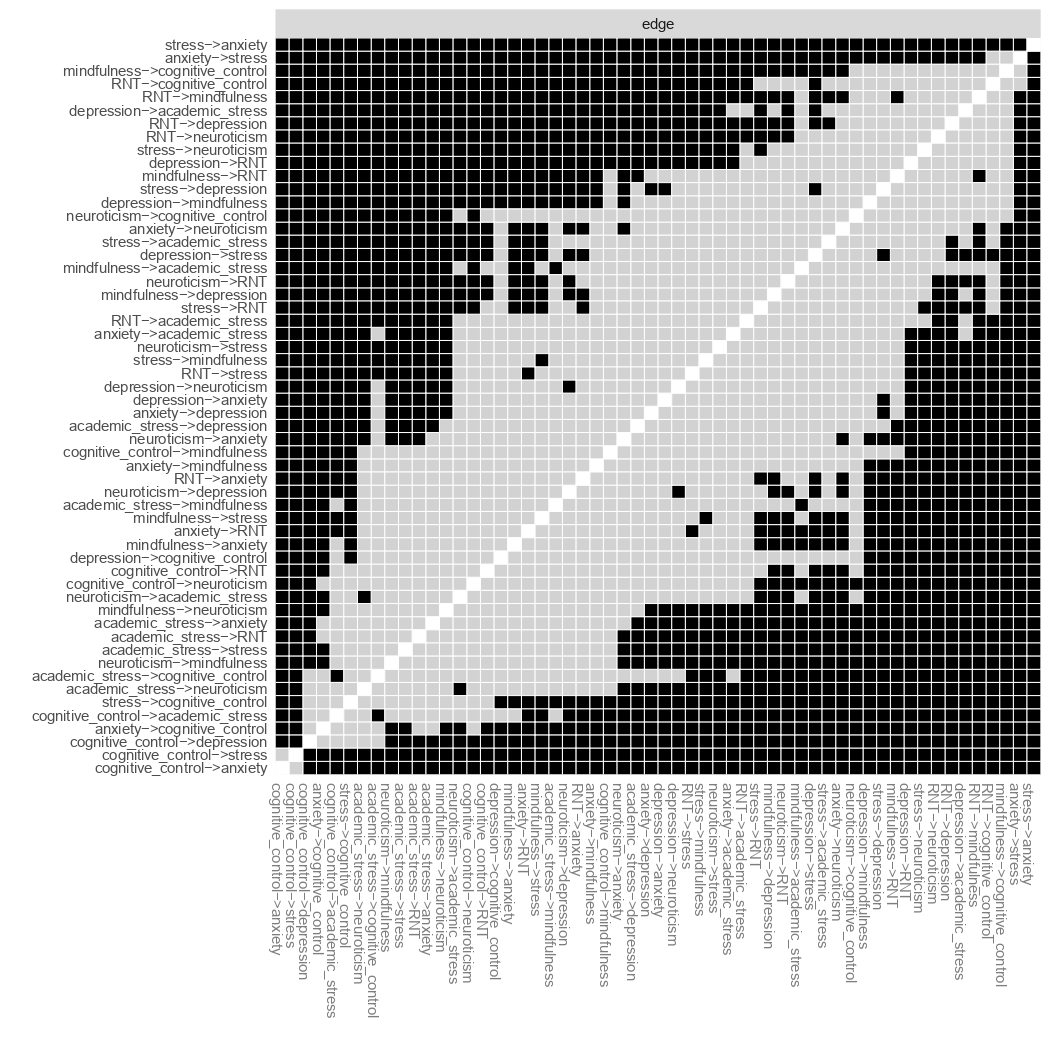


**Figure S8** Bootsrapped significance test of edge weights in the relative importance network. Note: A significant difference (p<.05) is represented by a black box at the intersection of a row and a column, while a grey box indicates no significant difference. ‘Stress’ refers to general stress (i.e., stress experienced in daily life, outside of academia) and ‘mindfulness’ connotes ‘mindful awareness.’
